# Perhydroazulene-based liquid-crystalline materials with smectic phases

**DOI:** 10.3762/bjoc.8.44

**Published:** 2012-03-16

**Authors:** Zakir Hussain, Henning Hopf, S Holger Eichhorn

**Affiliations:** 1 Institut für Organische Chemie, Technische Universität Braunschweig, Hagenring 30, 38106 Braunschweig, Germany, Fax: +49(531)3915388; 2 Department of Chemistry, COMSATS Institute of Information Technology, University Road, Abbottabad, Pakistan; 3 Department of Chemistry and Biochemistry, University of Windsor, 401 Sunset Avenue, Essex Hall, Windsor, ON Canada N9B 3P4, Fax: +1 (519) 973-7064

**Keywords:** hydroazulenes, liquid crystals, nematic phases, smectic phases

## Abstract

New liquid-crystalline materials with a perhydroazulene core were synthesized and the stereochemistry of these compounds was investigated. The mesomorphic properties of the new LC compounds were investigated by differential scanning colorimetry, polarizing optical microscopy and X-ray diffraction. We report here on the LC properties of nonchiral materials, which predominantly exhibit smectic phases and display nematic phases only within narrow temperature ranges. The dependence of the mesogenic behavior of the new materials on the stereochemistry of the core system was also investigated. All newly synthesized compounds were fully characterized by the usual spectroscopic and analytical methods.

## Introduction

Liquid crystals for display applications have to fulfill a complex, interdependent set of properties [1]. First of all, they must display a broad nematic phase, typically ranging from −30 °C to +80 °C. The absolute value for the dielectric anisotropy Δε should be large in order to decrease the operating voltage, since this will lower power consumption. The rotational viscosity γ_1_ should be as low as possible to allow fast switching, and the birefringence Δn has to be adjusted to fit the precise display configuration, in particular the cell gap. In the molecular design of new liquid crystals, electro-optical properties can now be predicted quite precisely based on molecular modeling [1–2]. However, full evaluation of the value of a new structure is still only possible after synthesis. In the past few years many attempts have been made to improve the properties of calamitic liquid crystals [3–8]. However, there is still a growing need for more advanced materials to be synthesized and tested for the desired features of the displays. Moreover, the properties of the LC materials required for applications in LCDs are achieved by mixture formulation of various components, including about 15–20 individual LC molecules.

The nematic (N) liquid crystal phase, with its orientational order only, is the most important mesophase; it is used in almost all commercially available LC displays. On the other hand, the smectic (Sm) LC phases, with their orientational order, have found little commercially successful applications. So far, an enormous amount of experimental and theoretical work exists in the literature concerning the nematic liquid crystals [1–8], whereas smectic liquid crystals have not been studied to the same extent, although recently they have attracted considerable interest [9–12]. It has also been demonstrated that by modification of the basic structures a wide range of properties affecting the liquid crystal behavior of these materials can be changed. The core units presently used in most of the calamitic LCs are cyclohexane, phenylcyclohexane, etc. We previously introduced [13] a completely new core unit: the perhydroazulene ring system. This system provides a segment that may align in such a way that the long molecular axes are parallel, with the capability to induce anisotropy, whereas if we introduce terminal chains, they can provide flexibility to stabilize the molecular alignment within the mesophase. Such terminal chains can either be nonpolar straight alkyl chains or carry a polar substituent. Such molecules may form both nematic and smectic mesophases depending upon the type of substituents and their substituent combinations, as well as on the stereochemistry at their central junction and the relative orientation of the respective substituents.

**Figure 1:**
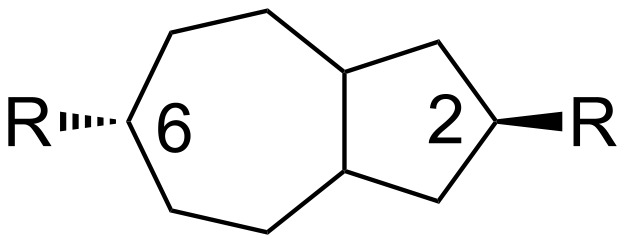
Overall molecular structure of the perhydroazulene core with *trans*-stereochemistry.

Hydroazulene-based liquid-crystal molecules are expected to show mesogenic behavior if linearity exists in the system. Such linearity can be achieved by the introduction of terminal groups preferentially in *trans*- or *anti*-fashion. We previously employed a ring expansion strategy for the synthesis of the perhydroazulene core and were able to also purify several of our carbene adducts by way of HPLC [14]. If we start our synthesis by the introduction of relevant terminal groups into the isomer that can provide the material with *trans*-stereochemistry, the resulting derivatives could reveal mesogenic behavior as expected (Figure 1).

In the present contribution, we report on the synthesis and mesogenic properties of materials containing a perhydroazulene core system and discuss the dependence of the LC properties of these materials on the stereochemistry of this core. Resulting from our investigations, we describe here that the perhydroazulene core system, having substituents with only *trans* orientation, provides mesogenic properties. To the best of our knowledge, such studies are so far absent from the literature with regard to hydroazulene-based smectic liquid-crystalline materials.

## Results and Discussion

**Scheme 1:**
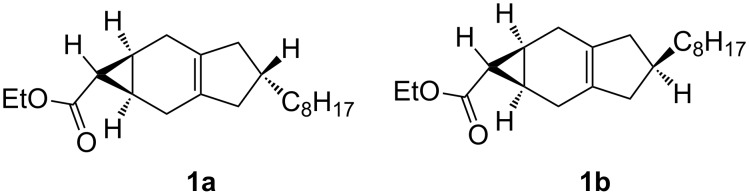
Stereochemistry of carbene adducts **1a** and **1b**.

The synthesis of *exo*-isomers **1a** and **1b** (Scheme 1) has already been reported [14]. Both isomers were purified through reversed-phase HPLC, obtained in >98% purity, and characterized by NMR spectroscopy as well as by their other analytical data [14]. The NMR data of **1a** and **1b** indicate that methylene groups in the five-membered ring carry pairwise enantiotopic and diastereotopic H-atoms; enantiotopic with respect to the internal mirror plane, diastereotopic with respect to above and below the plane of the molecules. A triplet at 1.40 ppm for **1a** and 1.34 ppm for **1b** with almost the same coupling constant (4.3 Hz) between the H-atoms at the cyclopropyl ring indicates an *anti-*relation between the ethoxycarbonyl group and the cyclohexene ring.

**Scheme 2:**
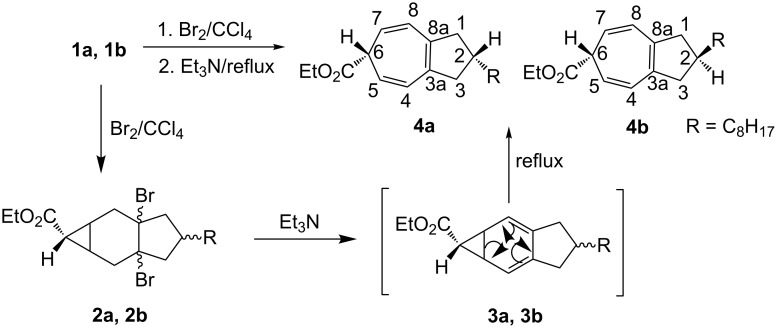
Preparation of the tropylidenes **4a** and **4b**.

The isomers **1a** and **1b** were subsequently subjected to a ring-opening reaction to afford the cycloheptatrienes **4a** and **4b**. In a first step, the isomers **1** were first brominated in carbon tetrachloride [15], to produce the dibromides **2a** and **2b**. After the complete addition of bromine, triethylamine was added slowly, resulting in triethylamine hydrobromide and the formation of the norcaradiene intermediates **3a** and **3b** in situ. These were subsequently converted by overnight heating into the cycloheptatrienes **4a** and **4b** as slightly colored liquids, by an electrocyclic ring-opening mechanism and a subsequent base-catalyzed prototropic shift, as described for analogous compounds [13]. In principle, the double bonds in the seven-membered rings of these derivatives could be arranged in a different fashion through the so-called Berson–Willcott rearrangement [16] or by 1,5-H shift processes. The exclusive generation of the particular isomers shown is presumably associated with their greater thermodynamic stability. With a double bond at the ring junction, the resulting isomer possesses the most highly substituted double bond possible (Scheme 2).

The two diastereomers **4a** and **4b** were separated and their structures were established by 1D and 2D NMR methods in addition to other analytical tools. The multiplet at 2.66 ppm in the ^1^H NMR spectrum for 6-H, doublets at 6.15–6.19 ppm with the coupling constant 9.1 Hz for 4/8-H and a singlet at 141.79 ppm in the ^13^C NMR spectrum for C-3a/8a indicated the formation of **4a** and **4b**. The orientation of the terminal groups and position of the double bonds in isomers similar to **4a** and **4b** was established by analysis of the X-ray structures of their corresponding acids [14].

**Scheme 3:**
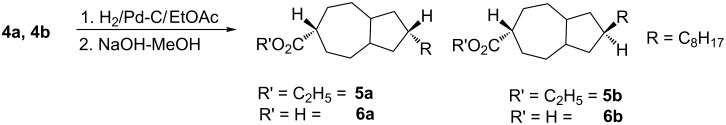
Formation of esters **5a** and **5b** and the corresponding acids **6a** and **6b**.

Continuing the synthesis, the precursors **4a** and **4b** were hydrogenated in the presence of Pd/C, and ethyl acetate as solvent which resulted in the completely saturated perhydroazulene systems **5a** and **5b**. It is important to note that, in addition to **5a** and **5b**, we also observed the presence of one additional isomer (<5%) in both cases in the GC-chromatogram. The formation of such an isomer from pure **4a** or **4b** can only be rationalized if catalytic hydrogenation results in an isomer with a *trans-*ring junction at the 3a/8a-position, as described previously [13–14]. However, in the present investigation, we could not completely purify this minor isomer in order to establish its exact stereochemical structure. Based on the full characterization of **4a** and **4b** by spectroscopic, analytical and X-ray data, the structures of the perhydroazulene systems **5a** and **5b** were elucidated. The disappearance of doublets at 6.1 ppm with coupling constants of 9.1 Hz for the 4/8 protons in **4a** and **4b**, and the appearance of multiplets for these protons, indicated the formation of **5a** and **5b**. Complete NMR (1D, 2D) as well as other analytical data of the above isomers can be found separately in the experimental section. In order to gain additional insight into the structures of the above esters (**5a**, **5b**) and to confirm their stereochemistry, their hydrolysis [17] was carried out in the presence of NaOH and MeOH. Acids **6a** and **6b** were obtained in the form of colorless solids, which were recrystallized from hexane/dichloromethane. Although the single crystals obtained were not suitable for a high-quality X-ray analysis, we could compare the NMR data of the resulting acids with the data of similar acids [13]. Based on this comparison, we established that the protons at 3a and 8a point in the same direction leading to *cis*-fused systems. The cycloheptane rings display very similar conformations, with local mirror symmetry through C6 and the midpoint of the C3a–C8a bond. Hydrogen bonding between the acids produces the usual dimeric forms. The complete analytical and spectroscopic data of **6a** and **6b** (Scheme 3) can be found in the experimental section.

**Scheme 4:**
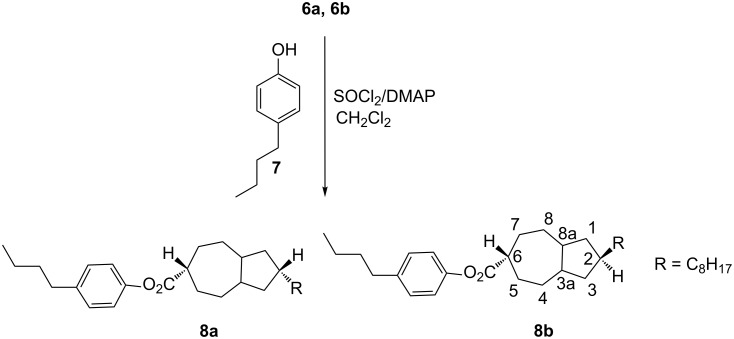
Preparation of **8a** and **8b**.

**Scheme 5:**
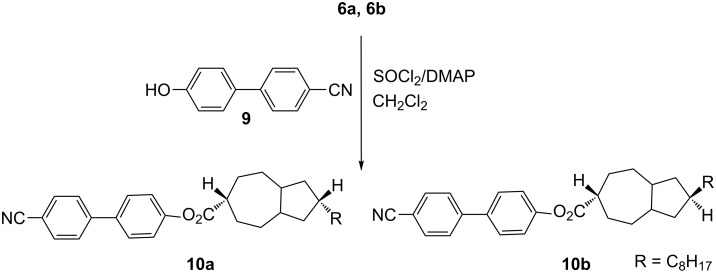
Preparation of **10a** and **10b**.

In a final step, esterification [18] of acids **6a** and **6b** was carried out in order to investigate the effect of stereochemistry on the liquid-crystalline properties in the resulting esters. We selected two different phenols for this esterification step, one with a nonpolar end group, an alkyl chain, and the other with a polar end group, a cyano moiety. The newly synthesized derivatives **8a** and **8b** (Scheme 4) and **10a** and **10b** (Scheme 5) were carefully purified and characterized through 1D and 2D NMR as well as through their other analytical data.

### Investigation of the mesogenic properties of the target compounds **8a**, **8b**, **10a** and **10b**

Investigation of the materials synthesized above for their phase-transition behavior showed that compounds **8a** and **10a**, with the ester and alkyl groups in 6- and 2-positions oriented *cis* to each other, do not display mesogenic behavior, and in both cases we see a clear crystal-to-isotropic transition (Table 1). However, compounds **8b** and **10b** with *trans-*orientations in the 6- and 2-positions showed several liquid-crystalline phases over a wide temperature range. Variable-temperature optical polarized microscopy (vt-OPM) and differential scanning calorimetry (DSC) measurements revealed that compound **8b** is converted into a SmC phase at ~28 °C and converts into a short-temperature-range SmA and N phase at ~101 °C and 107 °C, respectively. The conversion of the nematic phase to the isotropic phase in the case of **8b** was observed at 117 °C. Similar investigations on phase transitions of compound **10b** revealed that this material melts into a SmC phase at a much higher temperature, i.e., ~94 °C, compared to **8b**. This mesophase was stable up to 244 °C at which it converted into a short-range SmA phase followed by transitions into the nematic and isotropic phases at 253 °C and 266 °C, respectively. Appearance of transitions at much higher temperatures in case of **10b** compared to **8b** could be associated with the chemical structures of both compounds. In the case of **8b**, the presence of only one aromatic ring with a short alky chain at the ester side could result in a lower melting range, while the presence of cyanobiphenyl in the case of **10b** could cause its higher melting behavior.

**Table 1:** Liquid-crystal data of target materials **8a** and **8b** and **10a** and **10b**.

	Peak temperatures (enthalpies) of transitions on heating/cooling in °C (kJ/mol)					
	Cry→Cry	Cry→I	Cry→SmC	SmC→SmA	SmA→N	N→I

**8a**		41/30 (27.8/−27.4)				
**8b**	7.9^a^		28/7 (33.87/−24.28)	101/98 (1.63/−1.57)	107/104 (0.69/−0.92)	117/115 (0.48/−0.92)
**10a**		91/69 (19.72/−20.86)				
**10b**	77/58 (−5.87), 54 (−1.34), 52 (−0.72)		94/68 (13.54/−5.36)	244/222 (0.25/−0.005)	253/233 (0.15/−0.13)	266/263 (0.92/−0.52)

^a^Exothermic (cold crystallization) and endothermic transitions overlap (net enthalpic change is 5.04 kJ/mol).

## Conclusion

We have synthesized new liquid-crystalline materials with a perhydroazulene core and investigated their stereochemistry by advanced NMR spectroscopic methods. On the basis of the concept that linearity in these molecules results in liquid-crystalline phases, we present data here on smectic as well as nematic phases that are observed in isomers with only *trans*-positioned terminal groups. We have also shown that by changing terminal group(s), a broad range of LC phases results, characterized by the transition temperatures of smectic phases in those isomers that carry a polar terminal group compared to those with a nonpolar terminal group. We have also fully described the synthesis of acid **6b**, which is the key intermediate, from the starting material **1b** in three steps with a total average yield of about 72%.

## Experimental

TLC: Precoated plastic plates, PolyGram Sil G/UV_254_. Column chromatography: Silica gel 60 (70–230 mesh) Merck (Darmstadt). Mp below 200 °C: Büchi 510 melting-point apparatus, above 200 °C: Kofler-Heiztischmikroskop, uncorrected. ^1^H and ^13^C NMR: Bruker DRX-400, ^1^H NMR (400.1 MHz); ^13^C NMR (100.6 MHz); chemical shifts (δ) are expressed in parts per million (ppm) downfield from tetramethylsilane or by using the residual nondeuterated solvent as internal standards (CDCl_3_: ^1^H: δ 7.26; ^13^C: δ 77.00). IR: Nicolet 320 FT-IR and Bruker Tensor 27 spectrometer. Samples were prepared either as KBr pellets or as thin films. UV: In acetonitrile and methanol with a Beckman UV 5230 or a HP 8452A Diode Array spectrophotometer. MS: Finnigan MAT 8430 using the electron ionization method (EI, 70 eV). CH_2_Cl_2_ and Et_3_N were distilled from CaH_2_ under nitrogen, while all other chemicals were of reagent quality and used as obtained from the suppliers. Reactions were carried out under dried argon when necessary. The experimental procedures for the preparation of **4a, 4b**, **5a, 5b** as well as **6a, 6b** are essentially the same as described previously [13].

**Ethyl 2-octyl-1,2,3,6-tetrahydro-6-azulenecarboxylate (4a, 4b)**: Ethyl 4-octyl-1,1a,2,3,4,5,6,6a-octahydrocyclopropa[*f*]indene-1-carboxylate (**1a**, 0.8 g, 2.51 mmol) was dissolved in CCl_4_ (30 mL), and the solution was cooled in an ice bath. Bromine (0.41 g, 2.51 mmol) dissolved in CCl_4_ (5 mL) was added dropwise under stirring. When the addition was complete, triethylamine (1.12 g, 11.18 mmol) was added. Triethylamine hydrobromide began to form immediately. The mixture was heated under reflux for 18 h. After being cooled, the hydrobromide was filtered off. The filtrate was evaporated and the resulting oil partitioned between benzene and dilute aqueous acid (HCl). The benzene layer was washed with water and dried with MgSO_4_ and filtered to obtain the crude product. After solvent removal in vacuo, the product was purified by column chromatography on silica gel with dichloromethane and pentane (8:2) as eluents, to yield **4a** as light-bluish liquid (0.5 g, ~58%). Compound **4b,** also obtained as a light-bluish liquid (56% yield), was synthesized by the same procedure from **1b**.

**Compound 4a**: Bp 134–136 °C/5 Torr; ^1^H NMR (400.1 MHz, CDCl_3_) δ 0.86 (t, ^3^*J* = 7.07 Hz, 3H, 19-H), 1.28 (t, ^3^*J* = 7.14 Hz, 3H, 11-H), 1.22–1.26 (m, 14H, 12-H, 18-H), 2.21 (m, 1H, 2-H), 2.32–2.41/2.77–2.83 (m, 4H, 1-H, 3-H), 2.66 (m, 1H, 6-H), 4.25 (q, ^3^*J* = 7.13 Hz, 2H, 10-H), 5.32–5.39 (m, 2H, 5-H, 7-H), 6.15–6.19 (2 × d, ^3^*J* = 9.1 Hz, 2H, 4-H, 8-H); ^13^C NMR (100.6 MHz, CDCl_3_) δ 14.09/14.24 (2 × q, C-11, C-19), 22.67 (t, C-18), 28.34 (t, C-13), 29.32, 29.63, 29.80 (3 × t, C-14, C-16), 31.90 (t, C-17), 35.97 (t, C-12), 37.34 (d, C-2), 42.82 (2 × t, C-1, C-3), 45.22 (d, C-6), 60.92 (t, C-10), 115.99, 116.65 (2 × d, C-5, C-7), 125.31, 125.45 (2 × d, C-4, C-8), 141.79 (s, C-3a, C-8a), 173.24 (s, C-9); IR (film) 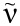: 2956 (m, CH-stretch), 173 (s, C=O), 1612 (w) cm^−1^; UV (CH_3_CN) λ_max_ nm (log ε): 228 (3.77), 236 (3.68), 246 (3.66), 264 (3.50); MS (EI, 70 eV) *m*/*z* (%): 316 (9) [M^+^], 243 (100) [M^+^ − C_3_H_5_O_2_], 129 (10) [243 − C_8_H_18_]; HRMS: calcd for C_21_H_32_NaO_2_, 339.229999; found, 339.22998 ± 0.07 ppm.

**Compound 4b:** Bp 135–137 °C/5 Torr; ^1^H NMR (400.1 MHz, CDCl_3_) δ 0.86 (t, ^3^*J* = 6.60 Hz, 3H, 19-H), 1.28 (t, ^3^*J* = 7.13 Hz, 3H, 11-H), 1.25–1.26 (m, 14H, 12-H, 18-H), 2.21 (m, 1H, 2-H), 2.31–2.41/2.77–2.83 (m, 4H, 1-H, 3-H), 2.68 (m, 1H, 6-H), 4.22 (q, ^3^*J* = 7.12 Hz, 2H, 10-H), 5.32–5.39 (m, 2H, 5-H, 7-H), 6.15–6.19 (2 × d, ^3^*J* = 9.13 Hz, 2H, 4-H, 8-H); ^13^C NMR (100.6 MHz, CDCl_3_) δ 14.08/14.23 (2 × q, C-11, C-19), 22.65 (t, C-18), 28.20 (t, C-13), 29.30, 29.62, 29.79 (3 × t, C-14, C-16), 31.88 (t, C-17), 36.16 (t, C-12), 37.79 (d, C-2), 42.99 (2 × t, C-1, C-3), 45.21 (d, C-6), 60.91 (t, C-10), 115.96, 116.63 (2 × d, C-5, C-7), 125.29, 125.44 (2 × d, C-4, C-8), 141.68 (s, C-3a, C-8a), 173.24 (s, C-9); IR (film) 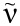: 2958 (m, CH-stretch), 1738 (s, C=O), 1610 (w), 1465 (w) cm^−1^; UV (CH_3_CN) λ_max_ nm (log ε): 228 (3.77), 236 (3.68), 246 (3.66), 264 (3.50); MS (EI, 70 eV) *m*/*z* (%): 316 (9) [M^+^], 243 (100) [M^+^ − C_3_H_5_O_2_], 129 (10) [243 − C_8_H_18_]; HRMS: calcd for C_21_H_32_NaO_2_, 339.229999; found, 339.23009 ± 0.27 ppm.

**Ethyl 2-octylperhydro-6-azulenecarboxylate (5a** and **5b):** Ethyl 2-octyl-1,2,3,6-tetrahydro-6-azulenecarboxylate (**4a**, 1.3 g, 4.11 mmol) was dissolved in ethyl acetate (100 mL) in a 250 mL flask and Pd/C (0.33 g) was added to the mixture. A stream of H_2_ was blown through the suspension after evacuation, and the flask was shaken for 2 h in a hydrogen atmosphere. The mixture was filtered to remove the catalyst and the solvent was evaporated to yield the product in quantitative yield as a colorless liquid. GC analysis indicated the presence of another isomer (about 15%), which could not be separated completely by column chromatography on silica gel, whereas the major isomer (>82%) was separated by elution with pentane, by increasing its polarity through the addition of dichloromethane (~50%). The major product was found to be the one with the *cis*-fused ring system, as expected (**5a**, 1.06 g, ~80%); this eluted first from the column followed by the isomer with the *trans*-fused system (always containing traces of the *cis*-fused derivative). Compound **5a:** Bp 145–150 °C/5 Torr; ^1^H NMR (400.1 MHz, CDCl_3_) δ 0.85 (t, ^3^*J* = 6.84 Hz, 3H, 19-H), 1.23 (t, ^3^*J* = 7.14 Hz, 3H, 14-H), 1.21–1.22 (m, 14H, side chain protons), 1.45–1.50 (m, 2H, 3a-H, 8a-H), 1.48–1.53 (m, 4H, 4-H, 8-H), 1.60–1.67 (m, 1H, 2-H), 0.82/1.81 (m, 4H, 1-H, 3-H), 2.00–2.09 (m, 4H, 5-H, 7-H), 2.57–2.62 (m, 1H, 6-H), 4.11 (q, ^3^*J* = 7.12 Hz, 2H, 10-H); ^13^C NMR (100.6 MHz, CDCl_3_) δ 14.09, 14.28 (2 × q, C-11, C-19), 22.67 (t, C-18), 28.73 (t, C-13), 28.79 (2 × t, C-4, C-8), 29.82 (2 × d, C-5, C-7), 29.33, 29.65, 29.97 (3 × t, C-14, C-15, C-16), 31.91 (t, C-17), 35.52 (t, C-12), 40.23 (d, C-2), 41.69 (2 × t, C-1, C-3), 42.12 (2 × t, C-3a, C-8a), 43.46 (d, C-6), 59.98 (t, C-10), 176.12 (s, C=O); IR (film) 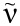: 2932 (s, CH-stretch), 1732 (s, C=O), 1463, 1450 (w) cm^−1^; UV (CH_3_CN) λ_max_ nm (log ε): 204 (2.92), 216 (2.55), 234 (2.15), 284 (1.86); MS (EI, 70 eV) *m*/*z* (%): 322 (100) [M^+^], 307 (17) [M^+^ − CH_3_], 293 (33) [M^+^ − C_2_H_5_], 276 (20) [M^+^ − C_2_H_6_O], 248 (16) [M^+^ − C_3_H_6_O_2_], 206 (55) [M^+^ − C_8_H_17_], 135 (42) [209 − C_3_H_6_O_2_]; HRMS: calcd for C_21_H_38_O_2_, 322.287178; found, 322.287430 ± 0.78 ppm.

**Compound 5b:** Compound **5b** was obtained from **4b** by the same procedure as described for **5a** in 77% yield as a colorless liquid. bp 147–151 °C/5 Torr; ^1^H NMR (400.1 MHz, CDCl_3_) δ 0.86 (t, ^3^*J* = 6.91 Hz, 3H, 19-H), 1.25 (t, ^3^*J* = 7.12 Hz, 3H, 14-H), 1.21–1.24 (m, 14H, side chain protons), 1.45–1.55 (m, 2H, 3a-H, 8a-H), 1.49 (m, 4H, 4-H, 8-H), 1.68 (m, 1H, 2-H), 0.86/1.92 (m, 4H, 1-H, 3-H), 2.13 (m, 4H, 5-H, 7-H), 2.55 (m, 1H, 6-H), 4.12 (q, ^3^*J* = 7.11 Hz, 2H, 10-H); ^13^C NMR (100.6 MHz, CDCl_3_) δ 14.11, 14.21 (2 × q, C-11, C-19), 22.67 (t, C-18), 28.73 (t, C-13), 28.86 (2 × t, C-4, C-8), 29.57 (2 × d, C-5, C-7), 29.33, 29.65, 29.97 (3 × t, C-14, C-15,C-16), 31.91 (t, C-17), 35.52 (t, C-12), 41.12 (d, C-2), 41.69 (2 × t, C-1, C-3), 41.90 (2 × t, C-3a, C-8a), 46.61 (d, C-6), 59.98 (t, C-10), 176.31 (s, C=O); IR (film) 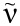: 2932 (s, CH-stretch), 1732 (s, C=O), 1463, 1450 (w) cm^−1^; UV (CH_3_CN) λ_max_ nm (log ε): 204 (2.92), 216 (2.55), 234 (2.15), 284 (1.86); MS (EI, 70 eV) *m*/*z* (%): 322 (100) [M^+^], 307 (18) [M^+^ − CH_3_], 293 (35) [M^+^ − C_2_H_5_], 276 (25) [M^+^ − C_2_H_6_O], 248 (10) [M^+^ − C_3_H_6_O_2_], 206 (53) [M^+^ − C_8_H_17_], 135 (45) [209 − C_3_H_6_O_2_]; HRMS: calcd for C_21_H_38_O_2_, 322.287176; found, 322.287435 ± 0.51 ppm.

**2-Octylperhydro-6-azulenecarboxylic acid (6a):** In a 250 mL round-bottomed flask EtOH (60 mL) and aqueous 1 M NaOH (36 mL) were placed. The solution was left to stir for a while and **5a** (300 mg, 0.93 mmol) was added slowly; the mixture was kept at room temperature and stirred for 4–6 h. When TLC analysis showed no more starting materials to be present, the mixture was acidified with 1 M HCl. The EtOH was evaporated and the residual aqueous solution was extracted twice with ethyl acetate and once with dichloromethane_._ The organic phases were combined, washed with water, and dried (MgSO_4_). The solvent was evaporated and the residue purified by column chromatography on silica gel by first eluting the impurities with dichloromethane and finally washing the column with Et_2_O to yield 240 mg (87%) of **6a** as a colorless solid. Acid **6b** was obtained from ester **5b** by the same procedure as for **6a** in 84% yield (230 mg), also as a colorless solid.

**Compound 6a**: Mp 102–104 °C; ^1^H NMR (400.1 MHz, CDCl_3_) δ 0.85 (t, ^3^*J* = 6.91 Hz, 3H, 17-H), 1.23 (m, 14H, side chain protons), 1.55 (m, 2H, 3a-H, 8a-H), 1.58 (m, 4H, 4-H, 8-H), 1.71 (m, 1H, 2-H), 0.78/2.22 (m, 4H, 1-H, 3-H), 2.15–2.58 (m, 4H, 5-H, 7-H), 2.69 (m, 1H, 6-H); ^13^C NMR (100.6 MHz, CDCl_3_) δ 14.11 (q, C-17), 22.66 (t, C-16), 28.72 (t, C-11), 29.41 (2 × t, C-4, C-8), 29.68, 29.90, 29.99 (3 × t, C-12, C-13, C-14), 31.08 (t, C-15), 31.93 (2 × t, C-5, C-7), 32.07 (t, C-10), 37.67 (d, C-2), 41.21 (2 × d, C-3a, C-8a), 41.43 (2 × t, C-1, C-3), 43.54 (d, C-6), 182.23 (s, C=O); MS (EI, 70 eV) *m*/*z* (%): 294 (40) [M^+^], 276 (18) [M^+^ − H_2_O], 265 (25) [M^+^ − C_2_H_5_], 181 (51) [M^+^ − C_8_H_17_], 179 (100) [181 − H_2_], 133 (52) [179 − H_2_CO_2_].

**Compound 6b:** Mp 103–106 °C; ^1^H NMR (400.1 MHz, CDCl_3_) δ 0.80 (t, ^3^*J* = 6.80 Hz, 3H, 17-H), 1.23 (m, 14H, side chain protons), 1.41–1.54 (m, 2H, 3a-H, 8a-H), 1.47–1.57 (m, 4H, 4-H, 8-H), 1.63–1.70 (m, 1H, 2-H), 0.67–0.70/1.83–2.10 (m, 4H, 1-H, 3-H), 2.15–2.58 (m, 4H, 5-H, 7-H), 2.67–2.69 (m, 1H, 6-H); ^13^C NMR (100.6 MHz, CDCl_3_) δ 14.09 (q, C-17), 22.67 (t, C-16), 28.72 (t, C-11), 29.35 (2 × t, C-4, C-8), 29.68, 29.90, 29.99 (3 × t, C-12, C-13, C-14), 31.08 (t, C-15), 31.93 (2 × t, C-5, C-7), 32.07 (t, C-10), 37.77 (d, C-2), 40.25 (2 × d, C-3a, C-8a), 42.48 (2 × t, C-1, C-3), 45.60 (d, C-6), 182.54 (s, C=O); MS (EI, 70 eV) *m*/*z* (%): 294 (36) [M^+^], 276 (14) [M^+^ − H_2_O], 265 (26) [M^+^ − C_2_H_5_], 181 (54) [M^+^ − C_8_H_17_], 179 (100) [181 − H_2_], 133 (52) [179 − H_2_CO_2_].

**General procedure for esterification of 8a, 8b and 10a, 10b**. Thionyl chloride (0.021 mL, 0.28 mmol) was added to a solution of 4-(*N*,*N*-dimethylamino)pyridine (DMAP, 34 mg, 0.27 mmol) in dichloromethane (5 mL) at −20 °C. Acid **6a** or **6b** (65 mg, 0.22 mmol) was added and the resulting solution was stirred for 1 h. Then, DMAP (34 mg, 0.27 mmol) and the phenol **7** (33.0 mg, 0.22 mmol) or **9** (43.0 mg, 0.22 mmol) were added and stirring was continued for another 1 h. The mixture was washed with water (5 mL) and the organic layer was separated and dried with sodium sulfate. The solvent was evaporated, and the remaining residue was separated by silica gel column chromatography, eluting with dichloromethane and pentane (1:1) to give the esters **8a**, **8b** or **10a**, **10b** as colorless solids.

**Compound 8a:** Mp 41–43 °C; ^1^H NMR (400.1 MHz, CDCl_3_) δ 0.86 (t, ^3^*J* = 6.95 Hz, 3H, 27-H), 0.90 (t, ^3^*J* = 7.35 Hz, 3H, 19-H), 1.24 (m, 14H, side chain protons), 1.30–1.35 (m, 2H, 17-H), 1.53–1.60 (m, 6H, 4-H, 8-H, 18-H), 1.63–1.66 (m, 1H, 2-H), 0.82/1.86 (m, 4H, 1-H, 3-H), 2.08–2.24 (m, 2H, 3a-H, 8a-H), 2.20–2.24 (m, 4H, 5-H, 7-H), 2.58 (t, ^3^*J* = 7.72 Hz, 2H, 16-H), 2.87–2.90 (m, 1H, 6-H), 6.93 (d, ^3^*J* = 11.10 Hz, 11-H, 15-H), 7.15 (d, ^3^*J* = 11.06 Hz, 2H, 12-H, 14-H); ^13^C NMR (100.6 MHz, CDCl_3_) δ 13.67, 13.87 (2 × q, C-19, C-27), 22.03 (t, C-18), 22.44 (t, C-26), 28.50 (t, C-21), 28.53 (2 × t, C-4, C-8), 29.47 (2 × t, C-5, C-7), 29.10, 29.41, 29.74 (3 × t, C-22, C-23, C-24), 31.68 (t, C-25), 33.37 (t, C-20), 34.78 (t, C-16), 35.28 (t, C-17), 40.03 (d, C-2), 41.52 (2 × t, C-1, C-3), 41.80 (2 × d, C-3a, C-8a), 43.30 (d, C-6), 120.97 (2 × d, C-11, C-15), 128.94 (2 × d, C-12, C-14), 139.91 (s, C-13), 148.62 (s, C-10), 174.53 (s, C=O); IR (film) 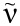: 2932 (s, CH-stretch), 1732 (s, C=O), 1463, 1450 (w), 1376, 1221 (w), 1183, 1143 (m), 1095, 1046, 1033 (w) cm^−1^; UV (CH_3_CN) λ_max_ nm (log ε): 204 (2.92), 216 (2.55), 234 (2.15), 284 (1.86), 312 (1.80), 342 (1.64), 374 (1.54); MS (EI, 70 eV) *m*/*z* (%): 426 (9) [M^+^], 276 (100) [M^+^ − C_10_H_14_O], 249 (17) [276 − CO], 163 (15) [276 − C_8_H_17_], 150 (85) [163 − CH]; HRMS: calcd for C_29_H_46_NaO_2_, 449.338997; found, 449.338715 ± 0.63 ppm.

**Compound 8b:** ^1^H NMR (400.1 MHz, CDCl_3_) δ 0.86 (t, ^3^*J* = 6.97 Hz, 3H, 27-H), 0.90 (t, ^3^*J* = 7.35 Hz, 3H, 19-H), 1.24 (m, 14H, side chain protons), 1.31–1.36 (m, 2H, 17-H), 1.51–1.58/1.70–1.74 (m, 6H, 4-H, 8-H, 18-H), 1.64–1.67 (m, 1H, 2-H), 0.77/1.97 (m, 4H, 1-H, 3-H), 2.08–2.13 (m, 2H, 3a-H, 8a-H), 1.53/2.22 (m, 4H, 5-H, 7-H), 2.46–2.52 (m, 1H, 6-H), 2.57 (t, ^3^*J* = 7.73 Hz, 2H, 16-H), 6.93 (d, ^3^*J* = 8.48 Hz, 11-H, 15-H), 7.13 (d, ^3^*J* = 8.50 Hz, 2H, 12-H, 14-H); ^13^C NMR (100.6 MHz, CDCl_3_) δ 13.69, 13.89 (2 × q, C-19, C-27), 22.07 (t, C-18), 22.46 (t, C-26), 28.50 (t, C-21), 29.12, 29.43, 29.76 (3 × t, C-22, C-23, C-24), 30.90 (2 × t, C-4, C-8), 31.70 (t, C-25), 32.02 (2 × t, C-5, C-7), 33.37 (t, C-20), 34.80 (t, C-17), 35.27 (t, C-16), 40.16 (d, C-2), 42.26 (2 × t, C-1, C-3), 42.86 (2 × d, C-3a, C-8a), 48.33 (d, C-6), 120.79 (2 × d, C-11, C-15), 128.84 (2 × d, C-12, C-14), 139.79 (s, C-13), 148.63 (s, C-10), 174.77 (s, C=O); IR (film) 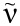: 2932 (s, CH-stretch), 1732 (s, C=O), 1463, 1450 (w), 1376, 1221 (w), 1183, 1143 (m), 1095, 1046, 1033 (w) cm^−1^; UV (CH_3_CN) λ_max_ nm (log ε): 204 (2.92), 216 (2.55), 234 (2.15), 284 (1.86), 312 (1.80), 342 (1.64), 374 (1.54); MS (EI, 70 eV) *m*/*z* (%): 426 (10) [M^+^], 276 (100) [M^+^ − C_10_H_14_O], 249 (21) [276 − CO], 163 (19) [276 − C_8_H_17_], 150 (85) [163 − CH]; HRMS: calcd for C_29_H_46_NaO_2_, 449.339001; found, 449.339077 ± 0.17 ppm.

**Compound 10a:** Mp 91–93 °C; ^1^H NMR (400.1 MHz, CDCl_3_) δ 0.86 (t, ^3^*J* = 6.97 Hz, 3H, 30-H), 1.24 (br m, 14H, side chain protons), 1.53 (br m, 4H, 4-H, 8-H), 1.66–1.69 (m, 1H, 2-H), 0.83/1.92 (m, 4H, 1-H, 3-H), 2.10–2.14 (m, 2H, 3a-H, 8a-H), 1.64/2.22 (m, 4H, 5-H, 7-H), 2.93–2.96 (m, 1H, 6-H), 7.17 (d, ^3^*J* = 8.73 Hz, 2H, 12-H, 14-H), 7.56 (d, ^3^*J* = 8.71 Hz, 2H, 11-H, 15-H), 7.64 (d, ^3^*J* = 8.68 Hz, 2H, 17-H, 21-H), 7.70 (d, ^3^*J* = 8.58 Hz, 2H, 18-H, 20-H); ^13^C NMR (100.6 MHz, CDCl_3_) δ 13.84 (q, C-30), 22.50 (t, C-29), 28.51 (t, C-24), 28.55 (2 × t, C-4, C-8), 29.12, 29.43, 29.75 (3 × t, C-25, C-26, C-27), 29.46 (2 × t, C-5, C-7), 31.70 (t, C-28), 35.30 (t, C-23), 40.06 (d, C-2), 41.54 (2 × t, C-1, C-3), 41.83 (2 × d, C-3a, C-8a), 43.45 (d, C-6), 110.44 (s, C-22), 118.70 (s, C-19), 122.16 (2 × d, C-11,C-15), 127.26 (2 × d, C-12, C-14), 128.00 (2 × d, C-17, C-21), 132.23 (2 × d, C-18, C-20), 136.48 (s, C-13), 144.65 (s, C-16), 151.16 (s, C-10), 174.29 (s, C=O); IR (film) 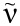: 2932 (s, CH-stretch), 1732 (s, C=O), 1463, 1450 (w), 1376, 1221 (w), 1183, 1143 (m), 1095, 1046, 1033 (w) cm^−1^; UV (CH_3_CN) λ_max_ nm (log ε): 204 (2.92), 216 (2.55), 234 (2.15), 284 (1.86), 312 (1.80), 342 (1.64), 374 (1.54); MS (EI, 70 eV) *m*/*z* (%): 471 (5) [M^+^], 276 (68) [M^+^ − C_13_H_9_NO], 249 (38) [276 − CO], 135 (49) [249 − C_8_H_18_], 109 (55) [135 − C_2_H_2_], 95 (100) [109 − CH_2_]; HRMS: calcd for C_32_H_41_NO_2_, 471.313732; found, 471.313327 ± 0.86 ppm.

**Compound 10b:** ^1^H NMR (400.1 MHz, CDCl_3_) δ 0.87 (t, ^3^*J* = 6.94 Hz, 3H, 30-H), 1.24 (br m, 14H, side chain protons), 1.33/1.77 (m, 4H, 4-H, 8-H), 1.65–1.69 (m, 1H, 2-H), 0.72/1.98 (m, 4H, 1-H, 3-H), 2.10–2.15 (m, 2H, 3a-H, 8a-H), 1.59/2.25 (m, 4H, 5-H, 7-H), 2.51–2.56 (m, 1H, 6-H), 7.14 (d, ^3^*J* = 8.70 Hz, 2H, 12-H, 14-H), 7.55 (d, ^3^*J* = 8.70 Hz, 2H, 11-H, 15-H), 7.63 (d, ^3^*J* = 8.54 Hz, 2H, 17-H, 21-H), 7.70 (d, ^3^*J* = 8.53 Hz, 2H, 18-H, 20-H); ^13^C NMR (100.6 MHz, CDCl_3_) δ 13.92 (q, C-30), 22.40 (t, C-29), 28.50 (t, C-24), 29.12, 29.43, 29.75 (3 × t, C-25, C-26, C-27), 30.90 (2 × t, C-4, C-8), 31.71 (t, C-28), 31.98 (2 × t, C-5, C-7), 35.27 (t, C-23), 40.01 (d, C-2), 42.32 (2 × t, C-1, C-3), 42.79 (2 × d, C-3a, C-8a), 48.31 (d, C-6), 110.66 (s, C-22), 118.62 (s, C-19), 122.03 (2 × d, C-11, C-15), 127.31 (2 × d, C-12, C-14), 127.96 (2 × d, C-17, C-21), 132.33 (2 × d, C-18, C-20), 136.55 (s, C-13), 144.55 (s, C-16), 151.16 (s, C-10), 174.54 (s, C=O); IR (film) 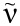: 2932 (s, CH-stretch), 1732 (s, C=O), 1463, 1450 (w), 1376, 1221 (w), 1183, 1143 (m), 1095, 1046, 1033 (w) cm^−1^; UV (CH_3_CN) λ_max_ nm (log ε): 204 (2.92), 216 (2.55), 234 (2.15), 284 (1.86), 312 (1.80), 342 (1.64), 374 (1.54); MS (EI, 70 eV) *m*/*z* (%): 471 (6) [M^+^], 276 (86) [M^+^ − C_13_H_9_NO], 249 (50) [276 − CO], 135 (56) [249 − C_8_H_18_], 109 (57) [135 − C_2_H_2_], 95 (100) [109 − CH_2_]; HRMS: calcd for C_32_H_41_NO_2_, 471.313726; found, 471.313482 ± 0.52 ppm.

## Supporting Information

File 1 Additional material.

## References

[1] Kirsch P, Bremer M (2000). Angew Chem, Int Ed.

[2] Klasen M, Bremer M, Götz A, Manabe A, Naemura S, Tarumi K (1998). Jpn J Appl Phys.

[3] Kirsch P, Reiffenrath V, Bremer M (1999). Synlett.

[4] Kelly S M (1991). Liq Cryst.

[5] Gray G W, Hird M, Lacey D, Toyne K J (1989). J Chem Soc, Perkin Trans 2.

[6] Miyazawa K, Kato T, Itoh M, Ushioda M (2002). Liq Cryst.

[7] Miyazawa K, de Meijere A (2001). Mol Cryst Liq Cryst.

[8] Demus D, Goto Y, Sawada S, Nakagawa E, Saito H, Tarao R (1995). Mol Cryst Liq Cryst.

[9] Cordoyiannis G, Sánchez-Ferrer A, Finkelmann H, Rožič B, Žumer S, Kutnjak Z (2010). Liq Cryst.

[10] Ema K, Yao H, Takanishi Y, Takezoe H, Kusumoto T, Hiyama T, Yoshizawa A (2002). Liq Cryst.

[11] Cordoyiannis G, Pinto L F V, Godinho M H, Glorieux C, Thoen J (2009). Phase Transitions.

[12] Gray G W (1987). Thermotropic Liquid Crystals.

[13] Hussain H, Hopf H, Pohl L, Oeser T, Fischer A K, Jones P G (2006). Eur J Org Chem.

[14] Hussain Z, Koley D, Hopf H (2005). Helv Chim Acta.

[15] Luhowy R, Keehn P M (1977). J Am Chem Soc.

[16] Berson J A, Willcott M R (1966). J Am Chem Soc.

[17] Luhowy R, Keehn P M (1976). Tetrahedron Lett.

[18] Arrieta A, Garcia T, Palomo C (1982). Synth Commun.

