# Supplementary material for: Perhydroazulene-based liquid-crystalline materials with smectic phases
Source: Beilstein J Org Chem. 2012 Mar 16;8:403–10. doi: 10.3762/bjoc.8.44 (PMC3326617; doi:10.3762/bjoc.8.44)
Supplement: File 1 — Additional material. [file Beilstein_J_Org_Chem-08-403-s001.pdf]

Supporting Information

for

Perhydroazulene-based liquid crystalline materials with smectic phases

Zakir Hussain,*[1,2] Henning Hopf[1], and S. Holger Eichhorn[3]

[1]Institut für Organische Chemie, Technische Universität Braunschweig, Hagenring 30, 38106 Braunschweig, Germany, Fax: +49(531)3915388, [2]Department of Chemistry, COMSATS Institute of Information Technology, University Road, Abbottabad, Pakistan and [3]Department of Chemistry and Biochemistry, University of Windsor, 401 Sunset Avenue, Essex Hall, Windsor, ON Canada N9B 3P4, Fax: +1 (519) 973-7064

Email: Zakir Hussain* - chem63@yahoo.com; Henning Hopf - H.Hopf@tu-bs.de; S. Holger Eichhorn - eichhorn@uwindsor.ca

* Corresponding author

Additional material

DSC curve of compound **10b** (heating/cooling rate 10 K/min)

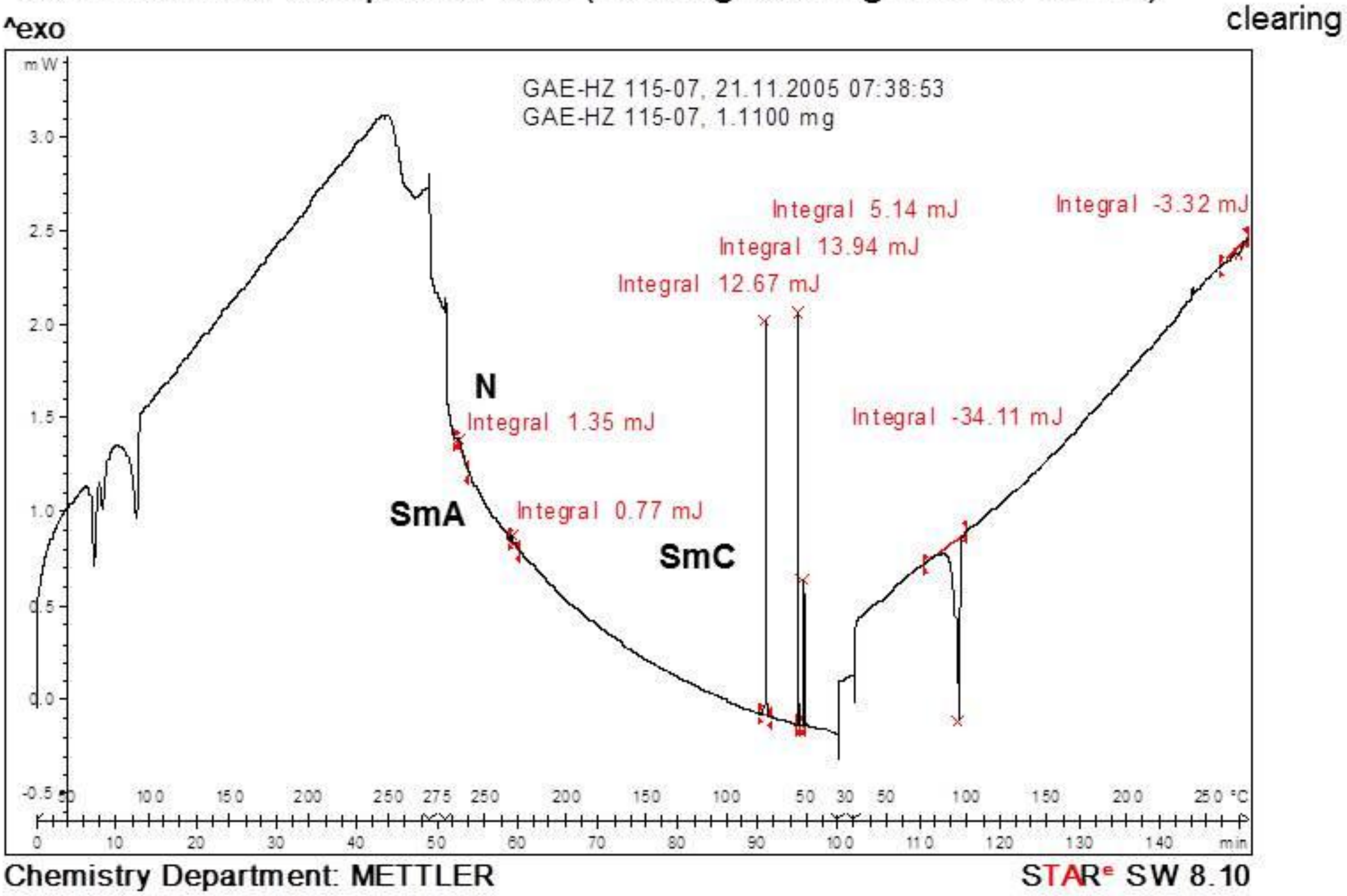

DSC curve of compound **10b** (heating/cooling rate 10 K/min)
magnified view with again 2 transitions on cooling

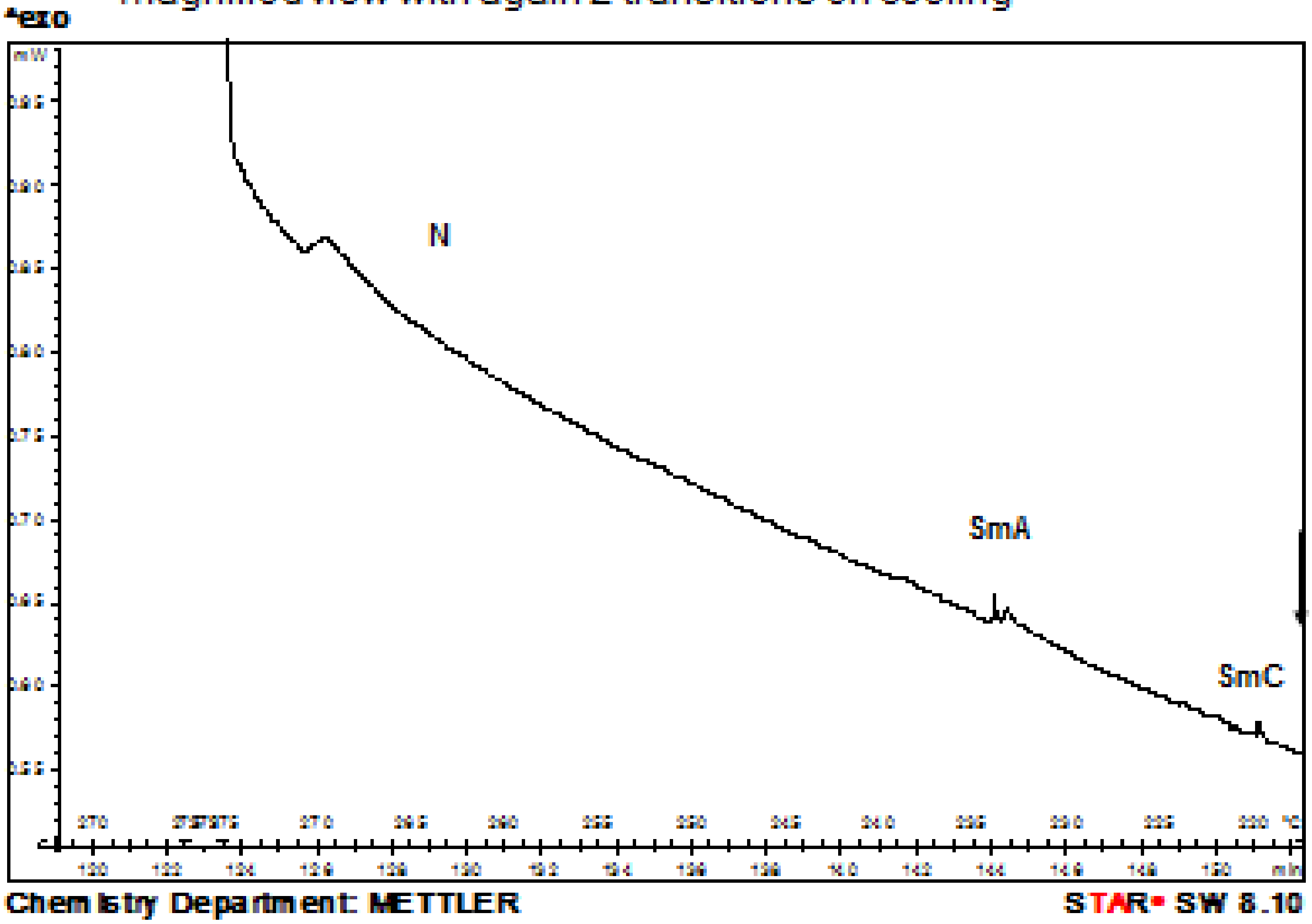

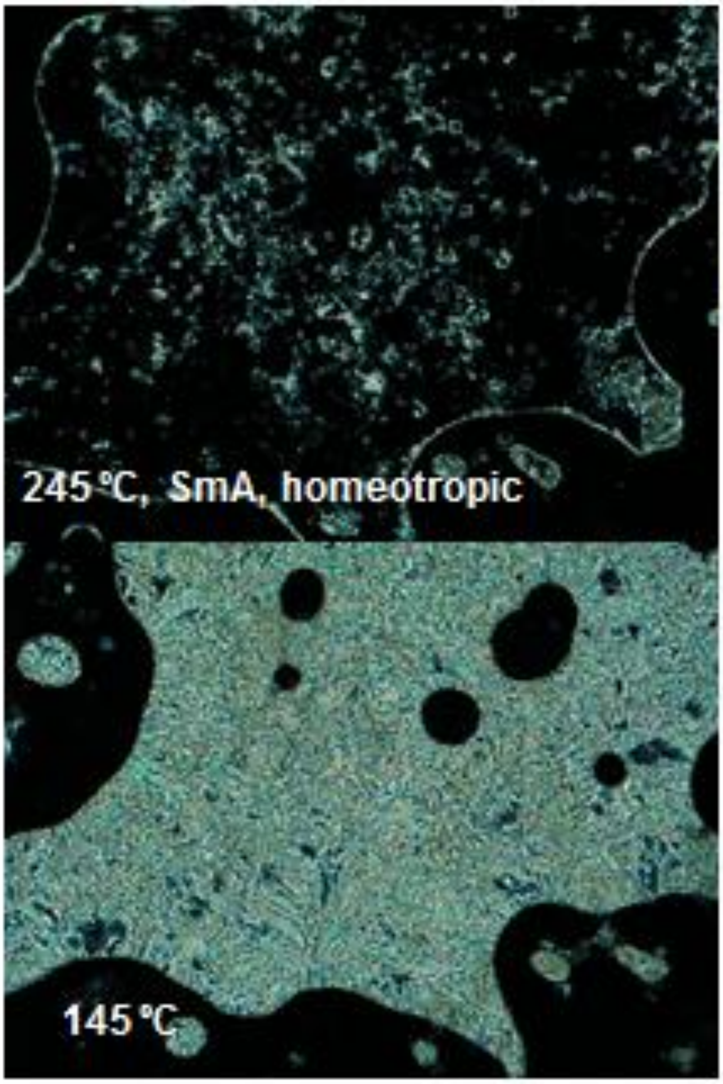

Textures of **10b** under crossed polarizers upon cooling from the isotropic liquid (magnification 200×, cooling rate 5 K/min) Temperatures for open stage, about 10 °C too high above 200 °C

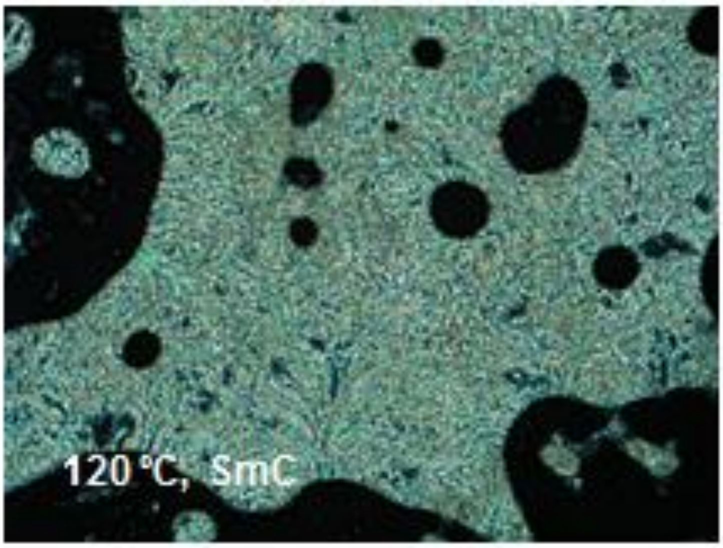

DSC curve of compound **8b** (heating/cooling rate 10 K/min)

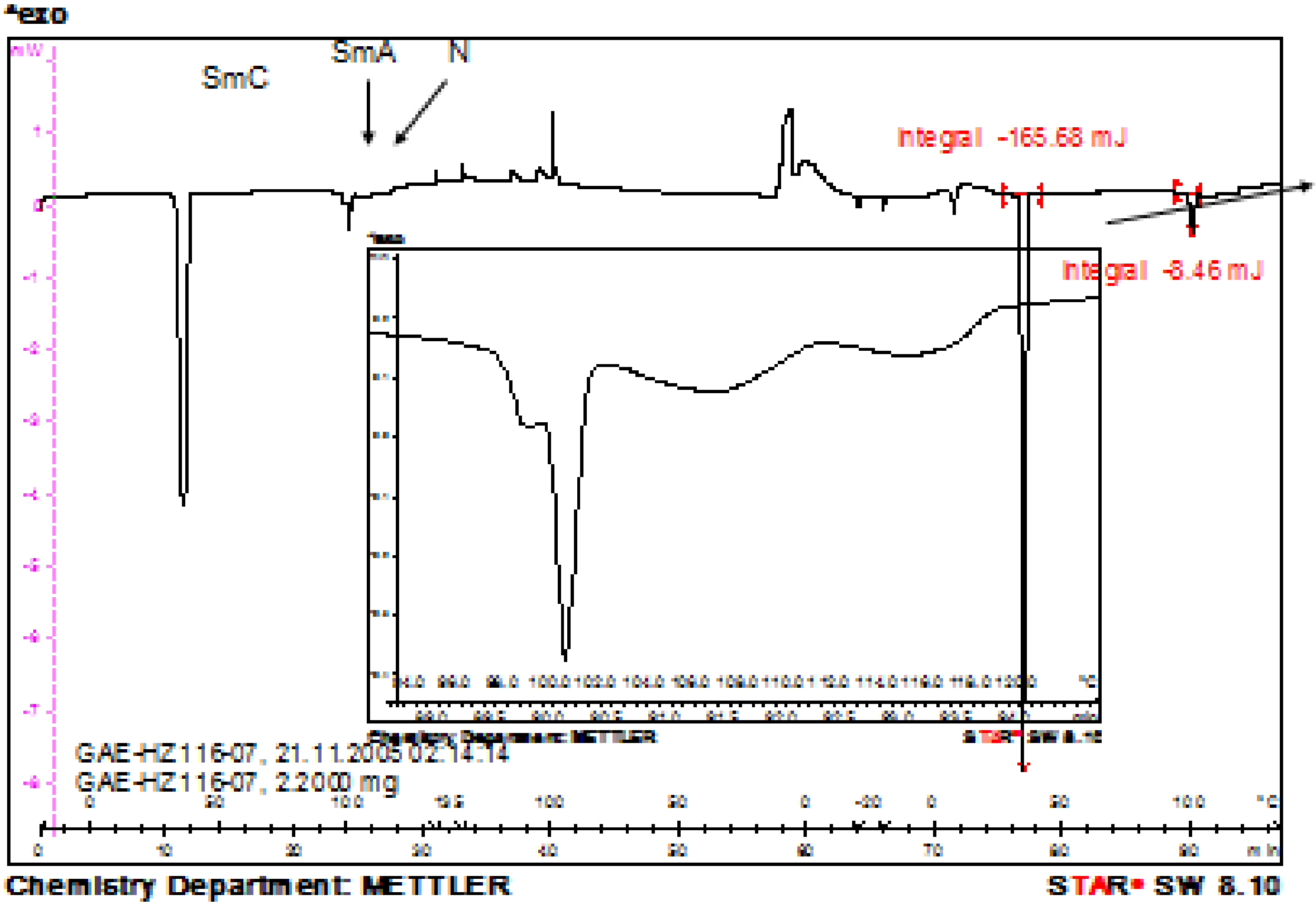

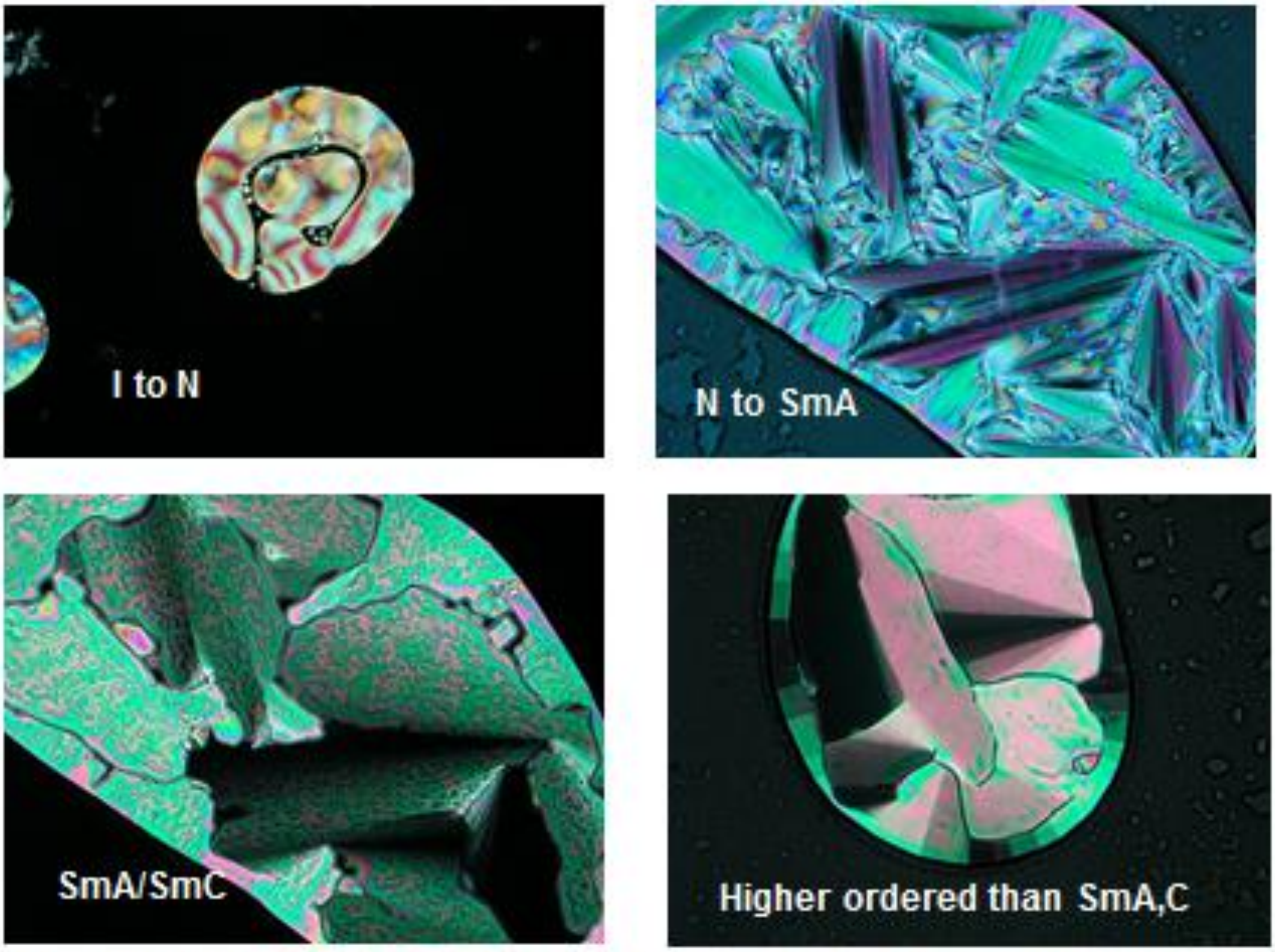

Textures of **8b** under crossed polarizers upon cooling from the isotropic liquid (magnification 200×, cooling rate 5 K/min)
